# Transactions of the Buffalo Obstetrical Society

**Published:** 1884-12

**Authors:** 


### TRANSACTIONS OF THE BUFFALO OBSTETRICAL SOCIETY.

*Stated Meeting, October 28, 1884.*

The President, Dr. W. W. Potter, in the Chair.

Dr. Stephen Y. Howell read a paper on "The Cæsarian Section." *

Dr. Lothrop, in opening the discussion, called attention to the great interest and importance that centers in this subject, as it was an operation in which two lives, though in the greatest jeopardy, could possibly be saved. Embryotomy was a horrible alternative. It was a question whether, in the present state of science, a physician could be justified in deliberately sacrificing

* See Original Communications, page 193.

the life of a child. The Cæsarian section, or some of its modifications, offers such strong probabilities of saving two lives that he considered it both unscientific and inhuman to perform in its stead any operation which inevitably sacrifices one. Thus, from a moral, as well as a scientific point of view, this operation commends itself to the obstetrician, and, he thought, must come more into vogue.

The Roman Catholic Church, and, also, the Anglican Church, as is well known, are opposed to the sacrifice of life without previous baptism; therefore, physicians of either faith could not conscientiously perform embryotomy; while the Cæsarian, or Dr. Thomas' operation, could be done without offense to any religious belief.

Dr. Lothrop thought well of Dr. Thomas' operation, laparo-elytrotomy, and, when feasible, believed it should stand first in the list. When, however, there was great deformity, it would be necessary, on account of the increased difficulty of extracting the child, to make, instead, the Cæsarian section or the Müller-Porro operation. He was inclined to give this latter the preference over the Cæsarian operation when laparo-elytrotomy could not be made, because of the lessened danger of hemorrhage.

Dr. Banta reported a case where the life of the child was presumably lost through neglect to make the post mortem Cæsarian section with the first instrument that came to hand. So much time was wasted in going for suitable instruments that the operation was useless. In future he would, in a similar case, lay open the abdomen with even the rudest of cutting instruments, and extract the child without delay. Coolness and dispatch must be united in him who would bring success from conditions where it seems so well-nigh impossible to triumph.

In regard to the Cæsarian section it behooves all physicians engaged in obstetric practice to bear its details well in mind—to hold its various modifications in regular order—so that when called into use there shall be no delay or hesitation. Procrastination is fatal to success. He considered embryotomy a most unjustifiable procedure from his standpoint. He was almost ready to say, in view of the advance that has been made, that it was unscientific as well, except only in extreme cases. He should not do it himself. He had performed it once, and, though the mother recovered, he has not, to this day, been forgiven by the family. He thought well of Thomas' operation, and considered it destined to lead all the others in the future, excepting in the class of extreme cases mentioned by Dr. Lothrop. In those cases, the essayist had presented the argument in favor of the Müller-Porro operation so strongly, that he was inclined to believe that it was the proper one to make.

Dr. Stockton wished to compliment the author upon the practical and attractive manner in which he had presented the subject. In order to relieve a woman in labor and save both her own life and that of her child, the natural passages being too small to allow the child to pass through, it is necessary to have a proper guide; in other words, to be well versed in the details of these various operations. The results presented by Dr Howell certainly merit serious consideration. There is no other test so accurate by which to judge an operation as its results. When by surgical interference you not only save the life of your patient, but also the life about to be brought into the world, both being certain to be lost if left to nature's efforts alone, you have achieved the greatest triumph in the field of obstetrics. Not the least of the factors entering into the success of the operation, described by the author of the paper, was the use of antiseptics, and, with their thorough and judicious employment, he predicted a great future for the Müller-Porro operation. He reminded the society that the late Prof. James P. White advised the performance of Cæsarian section whenever the natural passages were too small to give passage to the living child, and instructed his pupils most thoroughly in the various details of the operation.

Dr. Keene had, early in his professional career, pronounced for the Cæsarian section as against craniotomy. He had written a thesis on this subject, when a student, in which he took this ground, though this was before the days of antiseptic surgery, when the operation was not as practicable as now. Human life was sacred, and the physician should be very cautious in advising any operation that certainly destroyed it. In regard to the question of choice between the Müller-Porro operation and Cæsarian section, he would leave that to be decided by the exigencies of each case when presented. If prepared to do either operation, the particular circumstances surrounding each case furnish the only safe guide to a proper decision between the several methods.

A sound judgment could hardly go astray in making either form of the operation under discussion; but craniotomy, by all means, should be avoided.

Dr. Keene also gave due credit to the proper use of antiseptics, as contributing to the success of these operations in a very large measure. Before their employment the operations were only accidentally made, so to speak; but now, when we have command of the case, we can decide upon our plan of action beforehand, and arrange its details with a far greater certainty of success.

Dr. Ingraham, though he had never seen the operation performed, considered the Müller-Porro method most feasible, theoretically and practically. He would, by all means, advise it, in preference to any operation which inevitably destroyed the child. Therefore he would recommend the members to thoroughly familiarize themselves with the various steps of these several forms of operative delivery, in order that there might be prompt action when the emergency comes.

Dr. Hartwig was of the opinion that the old Cæsarian section had a future as well as the Müller-Porro operation. If it were possible to determine the precise locality of the placenta, the Cæsarian operation could be made absolutely bloodless, and thus the principal immediate danger, viz., hemorrhage, avoided. He proposed the following plan: After opening the abdomen down to the uterus, he would ascertain the position of the placenta, if possible, and if it was not found beneath the incision in the abdomen, strong stitches could be passed on each side of the abdominal incision through the uterine wall, so as to include nearly or quite its entire thickness in the sutures. These should then be drawn tight, which would securely fasten the uterine and abdominal walls to each other, while the incision through the uterus was being made, thus preventing hemorrhage. The membranes should then be ruptured, the child removed, and the cord tied, the placenta being left *in situ.* The wound in the uterus should next be closed by sutures, the stitches holding the uterus to the abdominal wall removed, and the womb allowed to contract and expel the placenta in the natural way.

Dr. H. had performed a post mortem Cæsarian section, but on account of the lapse of time after the death of the mother the child was found dead. It was interesting to note that the hemorrhage in this case was quite severe, notwithstanding the great length of time since the mother's death.

The President remarked that the unfavorable statistics of Cæsarian section were, undoubtedly, due to the fact that the earlier operations were chiefly those of emergency and not of selection. Given, a rachitic woman, or one with a justo-minor pelvis, with an outlet diameter too narrow to permit the passage of a living child, and he could see no reason, in these days of antiseptic surgery, why the statistics of the Cæsarian section may not be made to compare favorably with those of abdominal surgery in general. The profession is indebted to the labors of Dr. Robert P. Harris, of Philadelphia, for much recent light upon the Cæsarian operation. His papers had served to awaken interest in this somewhat neglected field.

He confessed to a preference for the Cæsarian section, though, since listening to Dr. Howell's excellent paper, he thought better of the Müller-Porro operation than ever before. In making Cæsarian section he would remove the placenta, and not leave it *in situ*, as proposed by Dr. Hartwig, even if he followed his plan in other respects. He considered Dr. Thomas' operation a most excellent expedient, when admissible, but regarded it of more limited application then either of the others. The Cæsarian section was the classical operation, having come down to us from a very remote period, though it was only now beginning to assume its proper place in obstetric surgery. He also reported a case in which he had recently made post mortem Cæsarian section, but without success, as he did not arrive until the mother had been too long dead.

Dr. Howell, in closing the discussion, wished to supplement his paper with a few remarks on pelvimetry. A very simple way of obtaining measurements of the pelvis, and one which he thought better than with any of the metallic pelvimeters, was by the fingers. Two fingers of one hand could be introduced into the pelvis as far as possible, and the extent of the introduction marked by the fingers of the other hand; then, withdrawing the fingers and subtracting three-fifths of an inch from the measure, will give the conjugate diameter very accurately.

He did not consider Dr. Thomas' operation of practical use. Even when undertaken, it has often to be abandoned, on account of the great difficulty of extracting the child, and the Cæsarian section substituted at greatly increased risk. Embryotomy was not justifiable, as the Cæsarian section, or some of its modifications, can be performed in every case without increased danger to the mother, and almost certain safety to the child.

---

